# The impact of probiotic supplementation on mucosal immunity and oxidative stress after high-intensity exercise among athletes: a meta-analysis

**DOI:** 10.1186/s13102-026-01746-1

**Published:** 2026-05-21

**Authors:** Chengguo Wang, Yang You, Jilan Zhou

**Affiliations:** Department of physical education, China University of Petroleum (East China), Huangdao District, Qingdao City, 266580 China

**Keywords:** Probiotic supplementation, High-intensity exercise, Mucosal immunity

## Abstract

**Objective:**

Athletes engaging in strenuous exercise can experience a heightened risk for upper respiratory symptoms and immune disorders triggered by exercise. This meta-analysis examined the relationship between probiotic supplementation, exercise, mucosal immunity, and oxidative stress (OS) levels following high-intensity exercise.

**Methods:**

We systematically searched four electronic databases to identify eligible studies for subsequent screening. The inclusion criteria are as follows: (1) Adults athletes; (2) Randomised Controlled Trial (RCT); (3) The experimental group received probiotic supplementation (Exp), while the control group received placebo (Con) supplementation; (4) Pre- and post-high-intensity exercise assessments of OS markers and salivary immunoglobulin A (sIgA) were conducted. Use the Cochrane bias risk assessment tool to evaluate the quality of the selected study. Select Standardized Mean Difference (SMD) as the appropriate effect scale index, and use Revman 5.4 software to analyze the mean difference of the selected article data.

**Results:**

A total of eight studies fulfilled the inclusion criteria and were selected for the meta-analysis. The results showed that the Exp group showed a significantly greater reduction in OS levels after high-intensity exercise compared to the Con group (SMD, -0.38 [-0.76, -0.01], *p* < 0.05, I^2^ = 0%). In addition, the Exp group showed a significantly greater improvement in sIgA levels compared to the Con group (SMD, 0.55 [0.09, 1.02], *p* < 0.05, I^2^ = 0%). Subgroup analysis showed that EXP group with more than 6 weeks significantly improved sIgA level (SMD, 0.78 [0.16, 1.41], *p* < 0.05, I^2^ = 0%), but not less than 6 weeks (SMD, 0.26 [-0.44, 0.96], *p* = 0.47, I^2^ = 0%).

**Conclusion:**

Probiotic supplementation, especially for more than 6 weeks, may enhance antioxidant capacity and mucosal immunity following high-intensity exercise.

**Supplementary Information:**

The online version contains supplementary material available at 10.1186/s13102-026-01746-1.

## Introduction

The highly competitive environment of professional sports necessitates that elite athletes continually push their boundaries, which is challenging amid a rapidly growing global population. Notably, athletes engaging in strenuous exercise can experience a heightened risk for upper respiratory symptoms and immune disorders triggered by exercise (cellular and humoral) [1]. These risks can be accompanied by acute and chronic alterations in secretory salivary immunoglobulin A (sIgA) [2]. Moreover, prolonged and intense exercise, coupled with insufficient or unsuitable nutrition, is generally linked to diminished immune cell function. This process can exacerbate the adverse effects of strenuous activity on immunocompetence [3]. Intense exercise and rigorous physical training also contribute to chronic fatigue and muscle damage, diminishing an athlete’s training efficiency. Hence, exercise-induced oxidative stress (OS) remains pivotal for repairing muscle, regeneration, and adapting redox signalling pathways. Sports physicians are also increasingly adopting a proactive strategy to prevent illness. This method can enhance recovery from fatigue, reduce training time lost due to illness, and improve athletic performance.

The possible impact of probiotics in mitigating and reducing fatigue recovery while decreasing the occurrence of upper respiratory symptoms within athletes is gaining popularity [4]. When consumed in sufficient quantities, probiotics are live microorganisms that provide health benefits to the host [5]. Specifically, four potential mechanisms of action may be primarily observed: (i) enhancement of the natural barrier function of the intestinal mucosa, (ii) regulating the immune response, (iii) inhibiting pathogens, and (iv) promoting enzymatic activities or beneficial metabolites for the host [6]. Probiotics also demonstrate antioxidant activity and can effectively mitigate systemic OS through various mechanisms [7]. These high antioxidant enzyme levels can directly neutralise oxidants in the intestinal tract [8]. In addition, an improved immune system can bolster mucosal immunity while mitigating cytokine-induced OS [9]. Limiting intestinal pathogens also reduces associated oxidative damage while enhancing immune function [10].

While existing meta-analyses [11] and reviews have explored probiotic effects on immune or oxidative stress markers in athletes separately, no prior meta-analysis has narrowly focused on the combined outcomes of OS and sIgA exclusively in athletes following high-intensity exercise—a critical research gap in the current literature. The correlation between probiotic supplementation, athlete mucosal immunity, and sIgA and OS levels after high-intensity exercise has not been fully resolved. Research has indicated that probiotic supplementation can enhance sIgA [12] and decrease OS levels following exercise in OS models [13]. Nevertheless, certain studies have suggested that probiotic supplementation does not significantly improve the above parameters [14, 15]. Thus, this review examined the relationship between probiotic supplementation, exercise, mucosal immunity, and OS levels following high-intensity exercise based on meta-analysis.

## Methods

### Study selection and data collection

This meta-analysis review protocol was officially recorded within the PROSPERO database (CRD420251004174) on 4th March 2025. Appendix A illustrates that two researchers conducted the search technique and manuscript drafting following the Preferred Reporting Items for Systematic Reviews and Meta-Analysis (PRISMA) standards. The search technique involved determining studies disseminated from 1st March 2006 to 1st March 2026 across four electronic databases: (i) EBSCO, (ii) PubMed, (iii) Scopus, and (iv) Web of Science. Several keywords were utilised for database searches, including “Probiotic*”, “Prebiotics”, “Synbiotic”, “Athlete*”, and “Player*.”

**Fig. 1:**
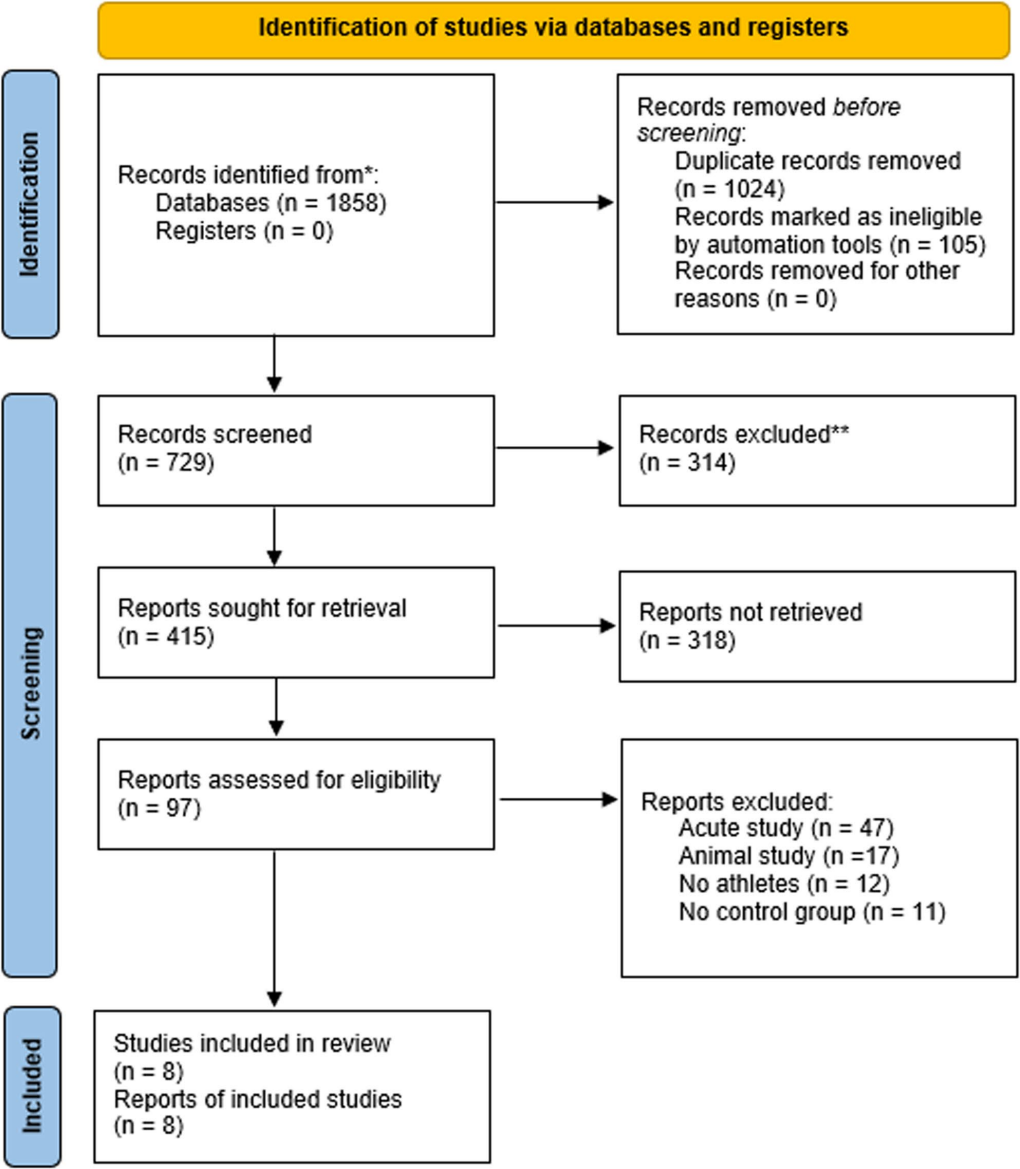
Flow diagram of the search results using the Preferred Reporting Items for Systematic Reviews and Meta-analysis (PRISMA)

Two independent investigators conducted the screening process utilising the titles and abstracts. The identified studies were re-evaluated by employing the inclusion and exclusion requirements established for this review. These two separate researchers also performed a quality examination while extracting data for studies that fulfilled the inclusion requirements. Data were then obtained from these studies. In disputes concerning the state of a document, a standalone researcher was consulted to provide input until an agreement was reached. Finally, the reference was obtained manually after confirming the inclusion of a document. Figure 1 depicts the document selection stages utilised in this review.

### Inclusion and exclusion criteria

The gathering of studies is conducted based on specific inclusion criteria as follows:

- Population: Respondents were athletes involved in competitive sports.
- Intervention: The experimental group (Exp) received probiotic supplementation.
- Comparison: In contrast, the control group (Con) received a placebo.
- Outcome: Analyses were performed pre- and post-training, focusing on OS following acute exercise and sIgA.
- Study design: Studies utilised the randomised controlled trial (RCT) approach.
- Other: Results were indicated as median (interquartile range) or mean ± standard deviation. Publication dates ranged from March 2006 to March 2026. Studies were published in English. The sole exclusion criterion for document selection in this review pertained to studies originating from grey literature.

### Quality assessment

The Grading of Recommendations Assessment, Development and Evaluation (GRADE) framework was used to assess the quality of each outcome estimate, with each quality rating categorized as high, moderate, low, or very low. These ratings were derived from scoring across five core domains: risk of bias, inconsistency, indirectness, imprecision, and additional considerations (Appendix B). The quality of the chosen studies was assessed utilising the Cochrane risk for determining the risk of bias [16]. Hence, the evaluated quality elements included blinding of outcome assessment, blinding of respondents and personnel, blinding of allocation, handling of missing data, generating random sequences, and selective reporting [17]. Every document received a score of “yes”, “no”, or “unclear.” Two researchers also separately examined the quality of every document. Conversely, a different researcher mediated any disagreements in the decision-making process.

### Data extraction

Information from each selected study was collected and organised into a table, encompassing numerous variables. These variables included age, gender, sports event, duration, probiotic protocol, placebo protocol, and index (Table 1). Two co-authors conducted data extraction independently, and a third researcher resolved disagreements.

**Table 1:** Characteristics of included studies

Study	Age and sample size (y; *n*)	Sex	Sport event	Duration	Probiotic	Placebo	Index
Gutierrez 2025	RPO: 33.0 ± 5.0;12 PLA: 32.0 ± 5.0;10	13 M 9 F	Multiple	4weeks, 7x/week	Capsules, Species: Lactobacillus plantarum, 10 × 10⁹ CFU/day.	Capsules, dextrose	GSH
Lamprecht 2012	RPO: 37.6 ± 4.7;11 PLA: 38.2 ± 4.4;12	23 M	Run	14weeks, 7x/week	Capsules, Species: Multi-strain probiotic containing Enterococcus faecium W54 etc. 10 × 10^10^ CFU/day.	Capsules, 4 g starch	PC
Macarro 2021	RPO: 25.3 ± 7.2;22 PLA: 27.1 ± 8.4;21	43 M	Cycle	6weeks, 7x/week	Capsules, Species: CECT 7347, CECT 9104 and CECT 8361, 1 × 10^9^ CFU/day.	Capsules, 300 mg maltodextrin and sucrose	MDA
Martarelli 2011	PRO: 32.0 ± 6.1;12 PLA: 32.0 ± 6.1;12	24 M	Cycle	4weeks, 7x/week	Capsules, Species: IMC 501 and IMC 502, 1 × 10^9^ CFU/day.	NA	ROM
Michalickova 2016	RPO: 22.5 ± 2.9;15 PLA: 23.6 ± 2.9;15	24M6F	Multiple	14weeks, 7x/week	Capsules, Species: L. helveticus Lafti L10, 2 × 10^10^ CFU /day.	Capsules, 1% magnesium stearate and 99% maltodextrin	sIgA
Pumpa 2019	RPO: 27.0 ± 3.2;9 PLA: 26.6 ± 2.9;10	19 M	Rugby	5weeks, 7x/week	Capsules, Species: Ultrabiotic 60™, 6 × 10^10^ CFU/day.	Capsules, microcrystalline, Iron, gelatin.	sIgA,
Quero 2019	RPO: 20.7 ± 1.39;7 PLA: 21.9 ± 2.8;6	13 M	Soccer	4weeks, 7x/week	Capsules, Species: CBP-001010, CNCM I-4036, ES1, 5 × 10^9^ CFU/day.	Capsules, 300 mg maltodextrin.	sIgA
Tavares-Silva 2021	RPO: 41.6 ± 3.2;7 PLA: 38.3 ± 3.1;7	14 M	Run	4weeks, 7x/week	Capsules, Species: LB-G80, LPc-G110, BB-G90, BL-G101, LLL-G25, 1 × 10^9^ CFU/day.	Capsules, 2.0 g corn starch	sIgA

*M* Male, *F *Female, *PRO *Probiotic group, *PLA* Placebo group, *NA*  Not Applicable, *CFU* Colony forming units, *GSH* Reduced glutathione and catalase, *PC* Protein carbonyls, *MDA* Malondialdehyde, *ROM* Reactive oxygen metabolites, *sIgA* Salivary Immunoglobulin A

### Data analysis

A meta-analysis was conducted utilising Review Manager (RevMan) software (v5.4.1, Copenhagen: The Nordic Cochrane Centre, Cochrane Collaboration, 2020) to evaluate the effects of the interventions. Given that the reviewed studies reported continuous variable outcomes using diverse testing methodologies, the standardised mean difference (SMD) was identified as the most appropriate effect size index. Nonetheless, the authors of the relevant studies were contacted via email for clarification in instances of undisclosed data.

A sensitivity examination was performed by systematically eliminating each document to evaluate the robustness of the meta-analysis outcomes if five studies or more exhibited a comparable measure. Based on previous research [18], the saliva flow rate of athletes may decrease by about 30% after high-intensity exercise. Therefore, this study also conducted sensitivity analysis by evaluating secretion rate. Heterogeneity in studies was also analysed using I^2^ statistics, in which an I^2^ close to zero suggests low heterogeneity among studies. Following standard criteria in meta-analyses, I² values were categorized as follows: <25% indicates low heterogeneity, 25–50% indicates moderate heterogeneity, and > 50% indicates high heterogeneity. This review also employed a random effect model for data analysis for the analysis [19]. Publication bias was assessed using a funnel plot (Figs. 5 and 6). Due to the small number of included studies per outcome (*n* < 10), Egger’s test was deemed unreliable and not conducted, acknowledging the limited statistical power for detecting publication bias. The uncertainty level was set at 95% CI.

## Results

### Eligibility of studies

Eight inclusion criteria were employed to conduct the systematic review. All studies included demonstrated ethical approval of their protocols from the relevant institutions. Two independent co-authors also presented a high level of consistency with one another (Cohen kappa coefficient = 0.94) throughout the screening phase. Among 473 participants reported in the selected studies, 175 were male, and 19 were female. A total of 97 patients were also assigned to the Exp group. Similarly, 97 patients were assigned to the Con group. The minimum and maximum intervention durations were four and 14 weeks, respectively.

### Quality assessment

**Fig. 2:**
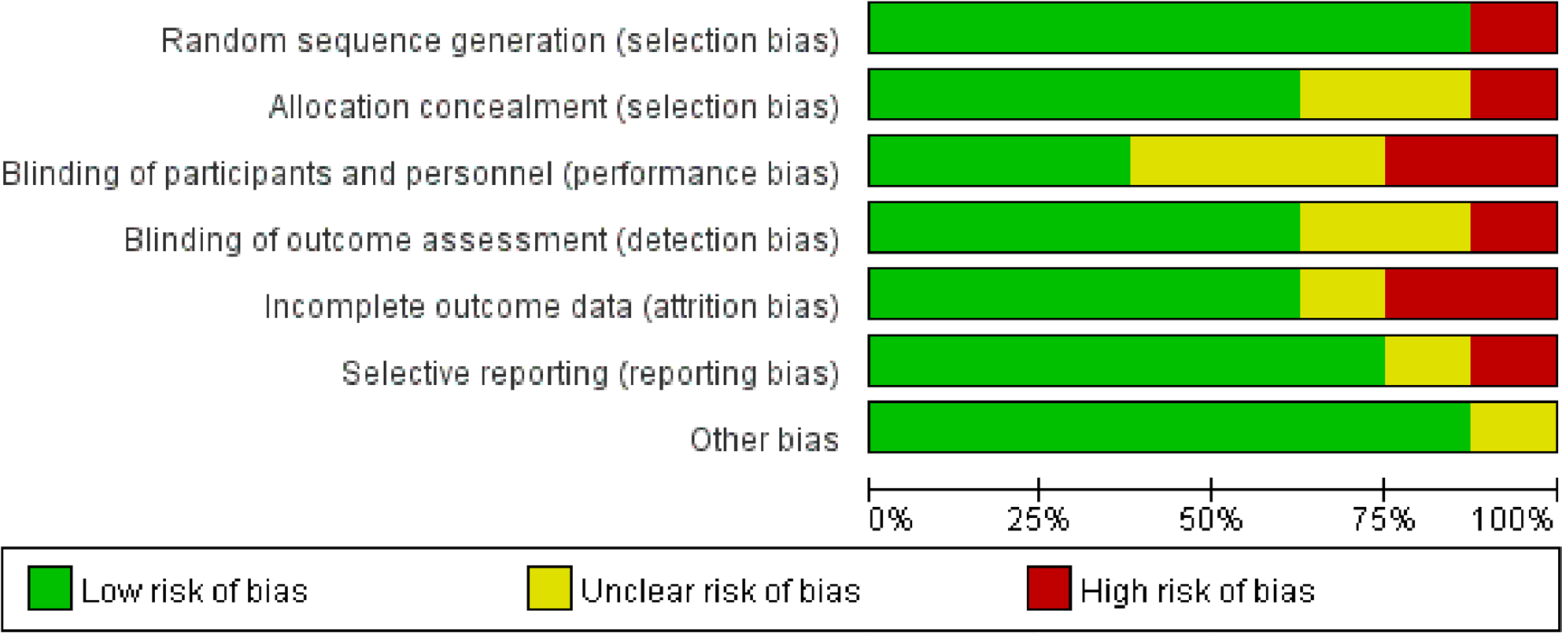
Analysis of risk of bias according to the Cochrane Collaboration guideline

The overall quality of the eight studies was significant, with a majority exhibiting a low risk of bias (Fig. 2). A small percentage of the studies also exhibited high bias, while the remaining articles were characterised by a lack of clarity. The summary table of various bias risks can be found in Appendix D.

### Quantitative synthesis

**Fig. 3:**
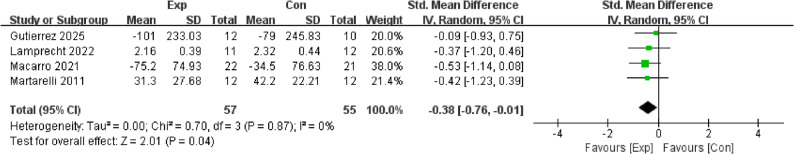
Forest plot portraying the effects of Exp vs. Con intervention on the oxidative stress

**Fig. 4:**
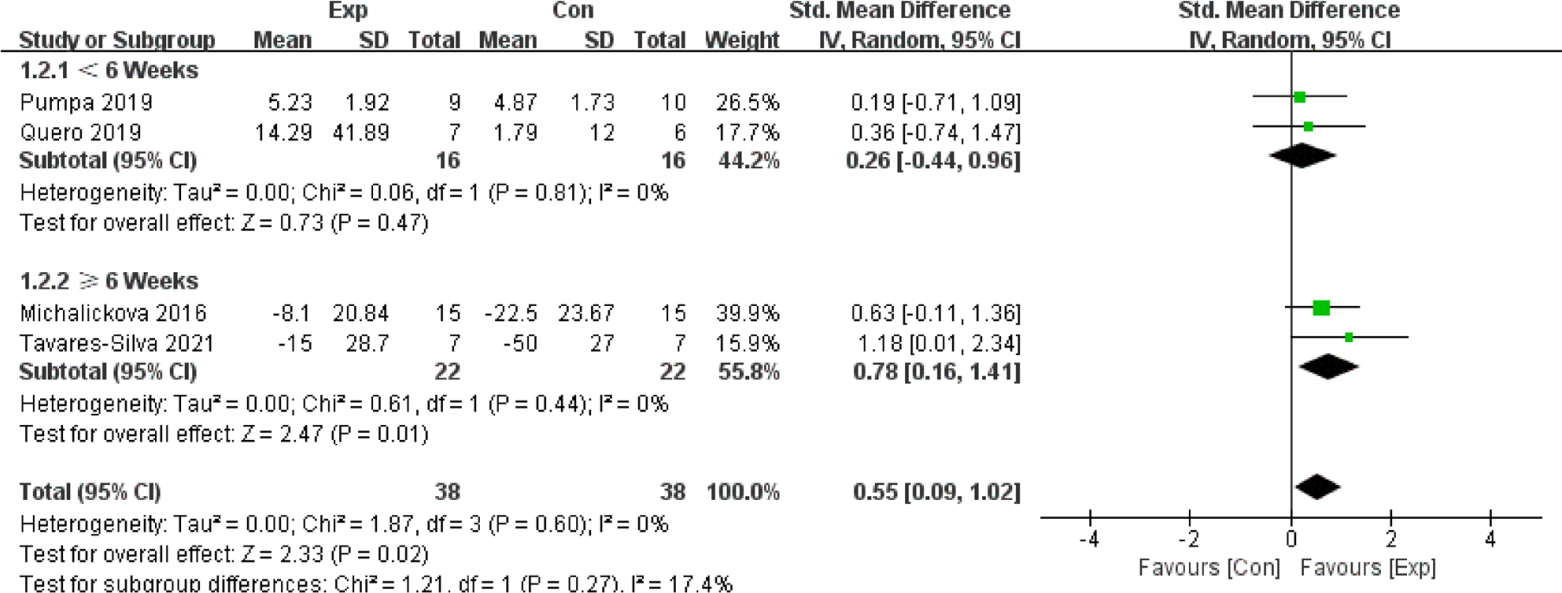
Forest plot portraying the effects of Exp vs. Con intervention on immunoglobulin A

The impact of the Exp and Con groups on OS was analysed across three included articles [13, 15, 20, 21] (Fig. 3). Consequently, the Exp group demonstrated a significantly greater reduction in OS levels after exercise and supplementation compared to the Con group (SMD, -0.38 [-0.76, -0.01], *p* < 0.05, I^2^ = 0%, *p* for heterogeneity = 0.87). Likewise, the effects of Exp and Con groups on sIgA were analysed in five included articles [12, 22–24] (Fig. 4). A meta-analysis further indicated that the Exp group had significantly higher sIgA levels compared to the Con group (SMD, 0.55 [0.09, 1.02], *p* < 0.05, I^2^ = 0%, *p* for heterogeneity = 0.60). Subgroup analysis showed that EXP group with more than 6 weeks significantly improved sIgA level (SMD, 0.78 [0.16, 1.41], *p* < 0.05, I^2^ = 0%, *p* for heterogeneity = 0.44), but not less than 6 weeks (SMD, 0.26 [-0.44, 0.96], *p* = 0.47, I^2^ = 0%, *p* for heterogeneity = 0.81).

### Publication bias analysis

**Fig. 5:**
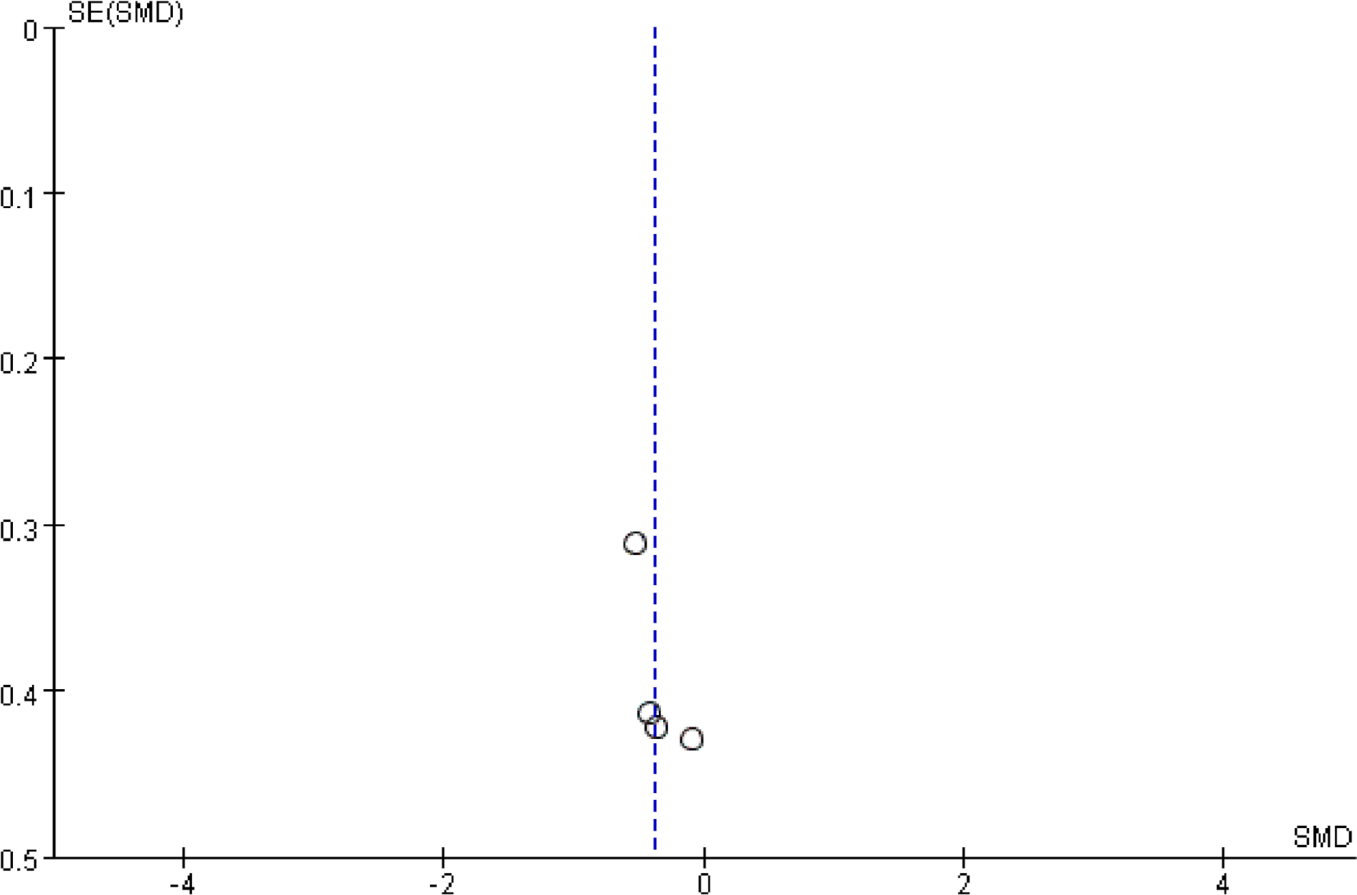
Funnel plot of publication bias in the Exp vs. Con intervention for the oxidative stress

**Fig. 6:**
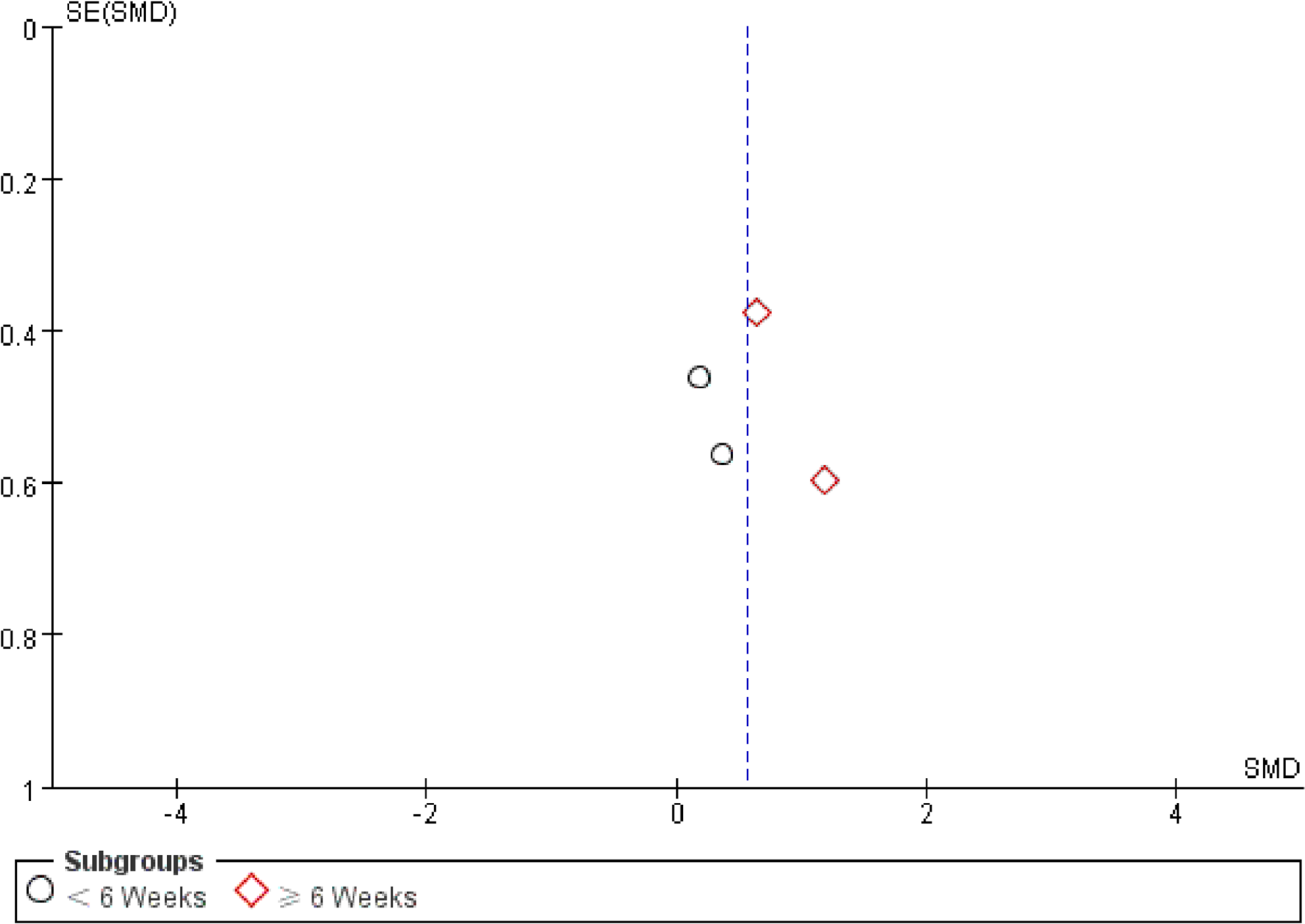
Funnel plot of publication bias in the Exp vs. Con intervention for the immunoglobulin A

The publication bias analysis indicated that eight articles met the minimum criteria of the funnel plot. Even though publication bias was suggestive to a limited degree, prior research indicated a minor likelihood of publication bias [25]. Figures 5 and 6 portray a left-right symmetrical, revealing a low probability of publication bias.

### Sensitivity analysis

The meta-analysis indicated no notable alterations within every group following modifications to the analysis type, effect size changes, and the exclusion of individual studies. Consequently, the sensitivity analysis indicated that the findings were reliable.

## Discussion

This review examined the influence of probiotic supplementation on the mucosal immunity of athletes and the acute post-exercise OS. Pooled evidence from the current meta-analysis suggests that probiotic supplementation may be associated with a moderate increase in sIgA levels (SMD = 0.55, 95% CI = 0.09 to 1.02) and a modest reduction in OS levels (SMD = -0.38, 95% CI = -0.76 to -0.01) compared with the placebo group. This outcome signified that probiotics could enhance athletes’ mucosal immunity while mitigating OS following high-intensity exercise when used as sports supplements.

Prebiotics, probiotics, and synbiotics have recently gained popularity as food supplements due to their positive effects on human health [26]. These products are due to their positive impacts, including pathogen suppression through competitive exclusion and immune system activation [27]. Considering that probiotics synthesise vitamins and engage in nutrient processing [28], their influence on probiotics towards host metabolism is notable. Particularly, several scenarios (poor nutrition, metabolic disorders, and antibiotic treatments) may necessitate probiotics to mitigate deficiencies in antioxidants and essential nutrients. Numerous reports have demonstrated that certain probiotic strains effectively mitigate OS following high-intensity exercise in athletes. This outcome is achieved through their antioxidative properties, enhanced food digestion and absorption, and immune system modulation [29, 30]. Furthermore, this review indicated that probiotics could prevent OS following high-intensity exercise.

Martarelli et al. [20] examined the effects of Lactobacillus rhamnosus IMC 501 and Lactobacillus paracasei IMC 502 supplementation on OS in athletes, with 12 participants in the experimental group receiving 4 weeks of probiotic intervention and 12 in the control group without supplementation. Consistent with our meta-analysis results, their study confirmed that intense physical activity induces OS, while probiotic supplementation increases plasma antioxidant capacity and neutralises ROS. Mechanistically, these two strains exert direct antioxidant activity by producing intracellular antioxidant enzymes and inhibiting linoleic acid peroxidation, a pathway that aligns with our observation of reduced OS levels in probiotic-supplemented athletes [20]. This consistency across individual studies and our meta-analysis strengthens the reliability of probiotics’ antioxidant effects in sports settings, addressing the prior lack of aggregated evidence for strain-specific efficacy.

Macarro et al. [13] reported comparable findings in which 22 male cyclists received probiotic supplementation for six weeks. The probiotics administered included *Bifidobacterium longum* 7347, *Lactobacillus casei* 9104, and *L. rhamnosus* 8361. Subsequently, the OS level was assessed before and following high-intensity exercise. The findings then indicated that the group receiving probiotic supplementation exhibited a significant increase in 8-hydroxy-2-deoxyguanosine, oxidized low density lipoprotein, and malondialdehyde levels compared to the Con group. This finding also supported the data of this review.

This review suggested a positive impact on sIgA. Generally, sIgA is consistently linked to infection incidence, with low concentrations or significant transient decreases correlating with an increased prevalence of upper respiratory tract diseases [31, 32]. Gleeson et al. [33] explained that regular intake of *L. casei* was advantageous in lowering the frequency of upper respiratory tract symptoms. This observation was attributed to the maintenance of sIgA levels in saliva, which was consistent with findings from other studies [31, 34]. The studies also indicated a significant finding, in which the enhancement of mucosal immunity through probiotic administration protected against infections caused by pathogens that breach the mucosa.

Michalickova et al. [23] randomly assigned 30 athletes to probiotic supplementation and placebo groups. The probiotic supplement was *Lactobacillus helveticus Lafti* L10, which was administered at a dosage of 2 × 10 [10] CFU daily. In contrast, the placebo group received an equivalent dosage of maltodextrin over 14 weeks. The findings indicated that probiotic supplementation significantly impacted sIgA more than the placebo group. This result was identical to the findings of this review. Nonetheless, the underlying mechanism by which probiotics may promote sIgA was not fully understood. This limitation was due to the high complexity of the sIgA-mediated immunity. Recent findings have also indicated that the creation of sIgA + B-cells occurs through T-cell dependent and T-cell independent. Nonetheless, the proportional impact remained ambiguous [35]. Mucosal sIgA+ cells typically exit the gut mucosa and enter the circulation, reaching local mucosal immune tissues (salivary glands). sIgA is produced and secreted into the salivary gland duct as sIgA [36].

Several limitations were observed in this review. Initially, the sample size was limited, encompassing only eight studies, and there was a significant sex imbalance among participants (89.7% male), which may limit the generalizability of the findings. In addition, the effects of probiotic supplementation were only investigated in athletic populations, with other populations largely excluded from consideration. Secondly, the included studies adopted various probiotic strains, leading to potential inconsistencies in the post-intervention effects; furthermore, none of the included studies reported measures of gut microbiota composition or abundance. Given that the core biological mechanisms of probiotic action are closely linked to the regulation of gut microbiota homeostasis, the absence of such data precludes an elucidation of the gut microbiota-mediated regulatory pathways underlying probiotics’ effects on mucosal immunity and oxidative stress in athletes. Due to the difficulty in obtaining detailed saliva flow rates for each study, sensitivity analysis was conducted to evaluate secretion rate parameters, taking into account the pre- and post-exercise change ratios proposed in previous research. Moreover, the differing research objectives across studies resulted in inconsistent evaluation schemes for athletes’ sports performance, limiting horizontal comparisons between individual studies. Hence, future studies should incorporate more relevant trials to facilitate a more comprehensive comparison of the effects of different probiotic strains; they should also integrate gut microbiota profiling with assessments of mucosal immunity and oxidative stress to clarify the mechanistic link between probiotic supplementation, gut microbiota modulation, and the observed immune and antioxidant effects in athletes. Additionally, determining the optimal probiotic dosage and the minimum effective dose is essential for enhancing athletes’ mucosal immunity and antioxidant capacity in the context of high-intensity exercise.

## Conclusion

Probiotic supplementation, especially for more than 6 weeks, may enhance antioxidant capacity and mucosal immunity following high-intensity exercise. Therefore, probiotics could positively influence athletes’ daily training status and were considered promising sports supplements.

## Supplementary Information

Supplementary Material 1.

Supplementary Material 2.

Supplementary Material 3.

Supplementary Material 4.

Supplementary Material 5.

## Data Availability

If necessary, it can be obtained from the corresponding author.
