# Supplementary material for: The impact of probiotic supplementation on mucosal immunity and oxidative stress after high-intensity exercise among athletes: a meta-analysis
Source: BMC Sports Sci Med Rehabil. 2026 May 21;18:351. doi: 10.1186/s13102-026-01746-1 (PMC13466530; doi:10.1186/s13102-026-01746-1)
Supplement: Supplementary file 1 [file 13102_2026_1746_MOESM1_ESM.docx]

**Appendix A**

Search strategy

| Databases | Search strategy | Result  (Approximately) |
| --- | --- | --- |
| Scopus | #1: Title-Abs-Key (Probiotic* or Synbiotic)  #2: Title-Abs-Key (Athlete* or Player*)  #4: #1 and #2 and #3  Limiters - Published Date: 20060301-20260315 | 73,127  198,421  547 |
| Pubmed | #1: [Title/Abstract] Probiotic* or Synbiotic  #2: [Title/Abstract] Athlete* or Player*  #3: #1 and #2  Filters: Publication date from 2016/03/01 to 2026/03/15 | 53,986  141,933  437 |
| Web of Science | #1: TOPIC: (Probiotic* or Synbiotic)  #2: TOPIC: (Athlete* or Player*)  #3: #1 and #2  Refined by: PUBLICATION YEARS: (20260315-20060301)  Indexes=SCI-EXPANDED, SSCI, CCR-EXPANDED, | 71,159  186,013  452 |
| EBSCO | #1: Abstract: (Probiotic* or Synbiotic)  #2: Abstract: (Athlete* or Player*)  #3: #1 and #2  Year: 20060301-20260315 | 67,271  134,171  422 |
