# Supplementary material for: The impact of probiotic supplementation on mucosal immunity and oxidative stress after high-intensity exercise among athletes: a meta-analysis
Source: BMC Sports Sci Med Rehabil. 2026 May 21;18:351. doi: 10.1186/s13102-026-01746-1 (PMC13466530; doi:10.1186/s13102-026-01746-1)
Supplement: Supplementary file 2 [file 13102_2026_1746_MOESM2_ESM.docx]

**Supplementary File 2 Grade evidence profile**

| **Certainty assessment** | | | | | | | **№ of patients** | | **Effect** | | **Certainty** | **Importance** |
| --- | --- | --- | --- | --- | --- | --- | --- | --- | --- | --- | --- | --- |
| **№ of studies** | **Study design** | **Risk of bias** | **Inconsistency** | **Indirectness** | **Imprecision** | **Other considerations** | **Theory-based interventions** | **Routine care** | **Relative (95% CI)** | **Absolute (95% CI)** |  |  |
| Oxidative stress | | | | | | | | | | | | |
| 3 | randomised trials | not serious | serious^b^ | not serious | serious^c^ | none | 45 | 45 | - | SMD **0.46 lower** (0.88 lower to 0.04 lower) | ⨁⨁◯◯ Low |  |
| Immunoglobulin A | | | | | | | | | | | | |
| 5 | randomised trials | not serious | not serious^b^ | not serious | serious^c^ | none | 52 | 52 | - | SMD **0.52 higher** (0.13 higher to 0.92 higher) | ⨁⨁⨁◯ Moderate |  |
| Salivary amylase | | | | | | | | | | | | |
| 2 | randomised trials | not serious | serious^b^ | not serious | serious^c^ | none | 23 | 24 | - | SMD **0.74 higher** (0.62 lower to 2.09 higher) | ⨁⨁◯◯  Low |  |

**CI:** confidence interval; **SMD:** standardised mean difference

a. Downgrade one level for most of the study have unclear or high risk of bias

b. Downgrade one level for the inconsistency due to considerable heterogeneity (I^2^ > 50%)

c. Downgrade one level for the imprecision due to the total sample size does not meet the rules of thumb (at least 400 participants)
