# Supplementary material for: The impact of probiotic supplementation on mucosal immunity and oxidative stress after high-intensity exercise among athletes: a meta-analysis
Source: BMC Sports Sci Med Rehabil. 2026 May 21;18:351. doi: 10.1186/s13102-026-01746-1 (PMC13466530; doi:10.1186/s13102-026-01746-1)
Supplement: Supplementary file 3 [file 13102_2026_1746_MOESM3_ESM.docx]

**Appendix D**

Table S1. Summary Table of Bias Risks.

| Study ID | Random sequence generation | Allocation concealment | Blinding of participants and personnel | Blinding of outcome assessment | Incomplete outcome data | Selective reporting | Other bias |
| --- | --- | --- | --- | --- | --- | --- | --- |
| Gutierrez 2025 | Low | Low | Unclear | Low | High | Low | Low |
| Lamprecht 2012 | Low | High | Unclear | Unclear | Low | High | Unclear |
| Macarro 2021 | Low | Low | Low | Low | High | Low | Low |
| Martarelli 2011 | Low | Low | High | Low | Low | Low | Low |
| Michalickova 2016 | Low | Unclear | High | Low | Low | Low | Low |
| Pumpa 2019 | High | Low | Unclear | Low | Low | Unclear | Low |
| Quero 2019 | Low | Low | Low | High | Unclear | Low | Low |
| Tavares-Silva 2021 | Low | Unclear | Low | Unclear | Low | Low | Low |
| Summary (n, %) | Low: 7 (88%); High: 1 (22%) | Low: 5 (63%); Unclear: 2 (25%); High: 1 (22%) | Low: 3 (48%); Unclear: 3 (48%); High: 2 (24%) | Low: 5 (63%); Unclear: 2 (25%); High: 1 (22%) | Low:5 (63%);  Unclear: 1 (22%); High: 2 (25%) | Low: 6 (75%); Unclear: 1 (25%);  High: 1 (25%) | Low: 7 (88%); Unclear: 1 (22%) |
