# Supplementary material for: The impact of probiotic supplementation on mucosal immunity and oxidative stress after high-intensity exercise among athletes: a meta-analysis
Source: BMC Sports Sci Med Rehabil. 2026 May 21;18:351. doi: 10.1186/s13102-026-01746-1 (PMC13466530; doi:10.1186/s13102-026-01746-1)
Supplement: Supplementary file 4 [file 13102_2026_1746_MOESM4_ESM.docx]

**Appendix C**

Figure S1. Forest plot statistics of sIgA secretion rate (Absolute concentration multiplied by saliva flow rate)
